# Overexpression of gene DEP domain containing 1 and its clinical prognostic significance in colorectal cancer

**DOI:** 10.1002/jcla.23634

**Published:** 2020-11-03

**Authors:** Xiaohan Shen, Jinming Han

**Affiliations:** ^1^ Ningbo Diagnostic Pathology Center (Shanghai Cancer Center Ningbo Pathology Center) Ningbo China; ^2^ Ningbo Medical Center Lihuili Hospital Ningbo China; ^3^ Ningbo NO. 6 Hospital Ningbo China

**Keywords:** biomarker, colorectal cancer, DEPDC1, prognosis

## Abstract

**Background:**

Colorectal cancer (CRC) is one of the most commonly seen malignancies worldwide, yet its regulatory mechanisms still need to be further illuminated. Abundant evidence revealed that aberrant expression of cancer‐related genes contributes to CRC progression. DEP domain containing 1 (DEPDC1) has been found to play a crucial role in the carcinogenesis and development of malignancies. Nevertheless, limited studies have been concerned with the role of DEPDC1 in CRC. This study aimed to investigate the relationship between DEPDC1 expression and CRC clinicopathological parameters.

**Methods:**

Solid CRC tissues and adjacent noncancerous tissues (ANCTs) (n = 150) were chosen randomly to detect the mRNA expression levels of DEPDC1 by real‐time quantitative reverse transcription‐polymerase chain reaction (RT‐qPCR). Formalin‐fixed, paraffin‐embedded (FFPE) blocks of CRC tissues and ANCTs (n = 150) were acquired to examine DEPDC1 protein expression levels by immunohistochemistry (IHC).

**Results:**

DEPDC1 was significantly overexpressed in CRC tissues than that in ANCTs (*P < .05*). High protein expression of DEPDC1 was associated with poorer TNM stage and recurrence (*P* < .001 and *P = *.003, respectively). Kaplan‐Meier survival analysis showed significantly shorter overall survival (OS) and disease‐free survival (DFS) in DEPDC1 protein high‐expression group compared with low‐expression group (*P < *.05). Univariate analysis demonstrated that DEPDC1 protein expression was correlated with DFS (*P = *.005) and OS (*P = *.006). Multivariate analysis revealed that the combination of DEPDC1 protein expression and TNM stage has statistical significance in CRC prognosis prediction (*P* = .024 and *P* = .009, respectively).

**Conclusions:**

DEPDC1 may act as a potential biomarker for CRC detection as well as a prognostic predictor concerning the survival of CRC patients.

## Graphical abstract

In total, 150 CRC patients were enrolled in the study. By IHC, we observed that the staining of DEPDC1 was in nucleus of CRC tumor cells. Representative immunostaining of DEPDC1 in CRC tissues and ANCTs (EnVision) are shown in Figures (A)‐(F): (A) CRC positive, weak (×100); (B) CRC positive, weak (×200); (C) CRC positive, strong (×100); (D) CRC positive, strong (×200); (E) ANCTs negative, (×100) (F) ANCTs negative, (×200). By RT‐qPCR, DEPDC1 was found significantly highly expressed in CRC tissues than in ANCTs (n = 150, *P < .05), shown in Figure (G). By Kaplan‐Meier analysis, DEPDC1 overexpression predicted poorer DFS and OS in CRC patients. (H) Relationship between DEPDC1 protein expression and DFS. (I) Relationship between DEPDC1protein expression and OS.

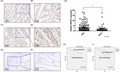

## 1 INTRODUCTION

Colorectal cancer (CRC) is one of the most commonly seen malignant tumors. In recent years, the burden of CRC disease has continued to increase worldwide.^1^, ^2^, ^3^, ^4^ According to latest statistics on the prevalence of malignant tumors in China, the incidence of CRC ranks 4th and 3rd among men and women, respectively, while the mortality ranks 5th among men and 4th among women.^2^ For many years, treatment plan and prognosis judgment of CRC have been continuously explored. With the advancement of radiotherapy and chemotherapy, molecular targeted biological therapy, and the increasing emphasis on early detection, the short‐term prognosis of CRC patients has been significantly improved, but the long‐term prognosis is still poor, and the 5‐year overall survival rate of advanced patients is still less than 20%.^3^ With the widespread application of molecular targeted drugs in the treatment of CRC, it is becoming more and more important to select appropriate markers for efficacy prediction. In recent years, the continuous development and clinical application of targeted drugs have effectively improved the prognosis of CRC patients, but the treatment effect of some patients is still unsatisfactory due to individual differences. Therefore, how to accurately screen effective populations through markers with predictive efficacy has become the focus and difficulty of targeted therapy.^5^, ^6^, ^7^ Many studies have shown that the development process of CRC is closely related to the expression or structural abnormality of a variety of oncogenes, tumor suppressor genes, and their products. Therefore, research on the molecular level to find CRC‐related targets can provide directions for predicting the efficacy of CRC targeted therapy and formulating precise treatment plans.^5^, ^8^, ^9^, ^10^

Human DEP domain containing 1 (DEPDC1), a gene containing a highly conserved DEP region, expresses related proteins containing DEP (namely Dishevelled, EGL‐10 and Pleckstrin) domain. The DEP domain is a protein sequence composed of nearly 100 amino acids. The protein containing this domain is involved in regulating various cellular functions, such as cell membrane anchoring, signal transduction, establishment of cell polarity, and regulation of small molecule GTPases.^11^, ^12^ Studies have found that DEPDC1 is associated with cell cycle progression, and abnormal expression of DEPDC1 can be observed in numerous malignant tumors including bladder cancer, breast cancer, and hepatocellular cancer (HCC).^11^, ^12^, ^13^, ^14^, ^15^, ^16^, ^17^, ^18^, ^19^ Nevertheless, the biological role of DEPDC1 in CRC remains hardly known. In our study, we aimed to detect the relationship between DEPDC1 expression levels and clinicopathological parameters to analyze whether this novel gene serves as a biomarker of CRC.

## 2 MATERIALS AND METHODS

### 2.1 Patient and tissue samples

All samples were obtained from Fudan University Shanghai Cancer Center. Formalin‐fixed, paraffin‐embedded (FFPE) blocks of CRC tissues (n = 150) and adjacent noncancerous tissues (ANCTs) (n = 150) were acquired from January 2011 to May 2013, along with available clinical‐pathological data. Fresh tissues from CRC patients were preserved in RNAlater (n = 150) in liquid nitrogen and stored in a − 80°C cryogenic freezer. The inclusion criteria were as follows: (a) adenocarcinoma; (b) age: 18‐75 years old; (c) staging: stage II‐III; (d) no preoperative chemotherapy or radiotherapy and targeted drug therapy before surgery; (e) no history of malignant tumors in other organs; (f) no family history mainly including familial adenomatous polyposis and hereditary non‐polyposis colorectal cancer; and (g) more than 5 years of follow‐up time. All CRC samples were independently assessed according to the WHO Classification of Tumours 5th Edition (2019) by two academic gastrointestinal pathologists to verify cancerous tissues and ANCTs. The data for clinicopathological parameters were collected from pathology diagnosis reports. This study was approved by the research ethics committee of Fudan University Shanghai Cancer Center, and the participants provided informed consent for the use of their tissues in this study.

### 2.2 RNA extraction and real‐time quantitative reverse transcription‐polymerase chain reaction

Total RNA was extracted from 150 CRC tissues and ANCTs using the TRIzol reagent (Invitrogen) following the instructions. A spectrophotometer (NanoDrop) and a Bioanalyzer (Agilent 2100) were utilized to evaluate the RNA concentration and purity, respectively. Based on 1 OD260 nm = 40 μg of RNA, the amount of RNA was tested, with an A260/A280 ratio from 1.8 to 2.1 as a criterion to qualify the RNA used in the experiments.^20^ First‐strand cDNA synthesis was performed by PrimeScript RT Master Mix (Sigma). The housekeeping gene GAPDH was utilized as an endogenous control. The cycling conditions for DEPDC1 and GAPDH were as follows: 95°C for 2 minutes (one cycle), followed by 95°C for 15 seconds (40 cycles), with the last extension at 60°C for 60 seconds. Dissolution curve analysis was performed after the cycle was over (95°C‐15 seconds; 60°C‐30 seconds; 95°C‐15 seconds). The gene DEPDC1 was amplified according to the following primers: 5′‐GCTACAAGTAAAGAGGGGATGG‐3′ (forward) and 5′‐GGACAGAAAGGTAAGTCAGTGGG‐3′ (reverse). The endogenous GAPDH gene was amplified according to the following primers: 5_‐ GCACCGTCAAGGCTGAGAAC (forward) and 5_‐ ATGGTGGTGAAGACGCCAGT‐3_ (reverse). Each measurement was repeated in triplicates. Gene expression in the 150 CRC tissues was examined by relative quantification method, with comparative cycle threshold (CT) (2 ^−ΔΔ^ *^C^* ^t^) method for relative gene expression.

### 2.3 Immunohistochemistry and evaluation of IHC staining

In total, 150 CRC tissues and ANCTs FFPE blocks were collected, and immunohistochemistry (IHC) assay was conducted using 4.5 μm paraffin sections. Sections were deparaffinized and rehydrated, then boiled with EDTA (pH 9) for approximately 10 minutes, and treated with 3% H_2_O_2_ in methanol for 30 minutes. After antigen recovery, 5% bovine serum albumin (BSA) was utilized for blocking non‐specific binding. Then, the slides were incubated at 4°C overnight with primary antibodies. Envision two‐step method was performed, and the DEPDC1 rabbit anti‐human polyclonal antibody (Sigma‐Aldrich) was utilized at a 1:400 dilution. CRC tissue slides with the incubation of primary antibody were utilized as negative controls.^21^ Immunostaining of the cell nucleus was scored.^12^ Scoring was performed following the previous description.^22^ Staining intensity was classified as 0‐3 scales: 0, absence; 1, weak; 2, moderate; and 3, strong. The percentage of positive tumor cells was scored as follows: 0, absence of tumor cells; 1, <33% positive tumor cells; 2, 33%‐66% positive tumor cells; and 3, >66% tumor cells. The final score was calculated by multiplying the staining intensity by the percentage score, ranging from 0 to 9. Scoring was conducted by two pathologists in a double‐blind manner. Differences larger than 10% need to be counted again.

### 2.4 Statistical analysis

In this study, SPSS 23.0 software was used for statistical analysis. On the basis of the mean of CRC tissue expression, data were listed as the means ± standard deviations for high or low DEPDC1 expression levels. Chi‐square test was used for the comparison of DEPDC1 protein expression between CRC tissues and ANCTs, as well as the relationships between clinicopathological characteristics and DEPDC1. Differences between measurement data were examined by Student's *t* test. Survival curves were plotted by Kaplan‐Meier method. Cox proportional hazard models (both Univariate and multivariate) were utilized to evaluate the correlations between CRC clinical characteristics and DEPDC1 expression. Values of *P < *.05 were defined as statistical significant.

## 3 RESULTS

### 3.1 DEPDC1 expression levels in CRC tissues and ANCTs

**Figure 1:**
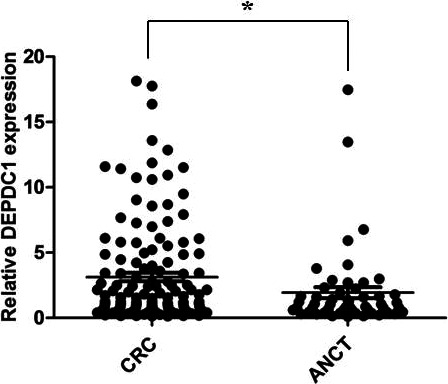
DEPDC1 was significantly highly expressed in CRC tissues than in ANCTs (n = 150, *P < .05). ANCTs, adjacent noncancerous tissues; CRC, Colorectal cancer

**Figure 2:**
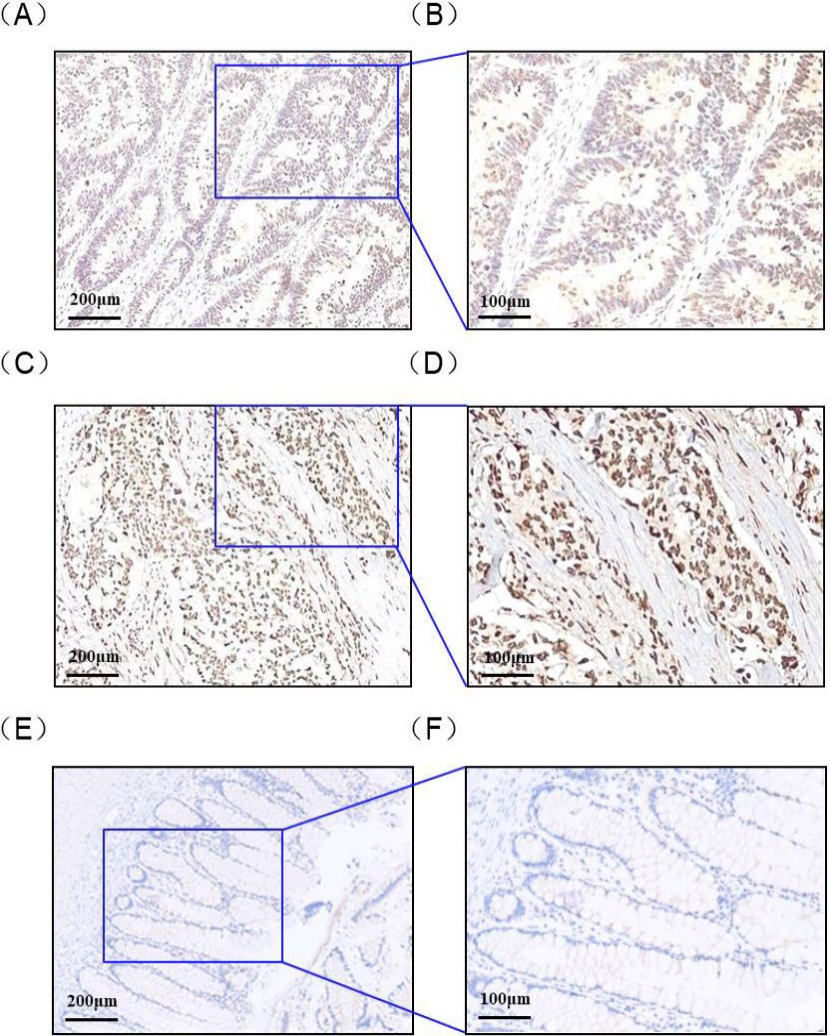
Representative immunostaining of DEPDC1 in CRC tissues and ANCTs (EnVision). (A) CRC positive, weak; (×100). (B) CRC positive, weak; (×200). (C) CRC positive, strong; (×100). (D) CRC positive, strong; (×200) (E) ANCTs negative; (×100). (F) ANCTs negative; (×200). ANCTs, adjacent noncancerous tissues; CRC, Colorectal cancer

We investigated whether DEPDC1 expression is detectable and different in CRC tissues compared with ANCTs. In total, 150 CRC patients were enrolled in the study. RT‐qPCR was performed with RNAs of CRC tissue samples to examine DEPDC1 mRNA expression levels. Statistics showed that median relative expression of DEPDC1 in CRC tissues and ANCTs was 3.233 and 2.112, respectively; 95% confidence interval (CI) of the difference: lower, 1.465; upper, 2.601. The results manifested that DEPDC1 mRNA expression was significantly higher in CRC tissues than that in ANCTs (*P <* .05, Figure 1). Next, we performed IHC to detect the protein expression of DEPDC1 in 150 CRC tissue samples and ANCTs. We observed that the staining of DEPDC1 was in nucleus of CRC tumor cells (Figure 2A‐F). The proportion of DEPDC1 high expression was 72.0% (108/150), while the number was 28.0% (42/150) in DEPDC1 low‐expression group, compared to 38.0% (57/150) and 62.0% (93/150) in ANCTs, respectively, which shows a statistical significance between the DEPDC1 protein expression of CRC and ANCTs (*P *= .019, Table 1).

**Table 1:** Comparison of DEPDC1 protein expression in CRC tissues and ANCTs

Sample types	No.	DEPDC1 expression		χ2	*P*
		High (n%)	Low (n%)		
CRC	150	108 (72.0)	42 (28.0)	5.542	.019
ANCTs	150	57 (38.0)	93 (62.0)		

Abbreviations: ANCTs, adjacent noncancerous tissues; CRC, Colorectal cancer.

### 3.2 DEPDC1 expression and clinicopathological parameters of CRC

Then, we assessed the association between the DEPDC1 protein expression and clinicopathological factors. As shown in Table 2, elevated DEPDC1 protein expression was correlated with poorer TNM stage and recurrence (*P *< .001 and *P *= .003, respectively). There was no significant correlation between DEPDC1 protein expression and other clinicopathological parameters, such as age, gender, tumor location, tumor size, differentiation, or state of life (*P *> .05, Table 2).

**Table 2:** Relationship between DEPDC1 protein expression and CRC patient clinicopathological parameters

Clinicopathological parameters	No.	DEPDC1 expression		*P*
		Low (42)	High (108)	
Gender				
Male	87	21	66	.216
Female	63	21	42	
Age (y)				
<60	89	23	66	.477
≥60	61	19	42	
Tumor size (diameter)				
<5 cm	92	25	67	.777
≥5 cm	58	17	41	
Location				
Colon	99	29	70	.623
Rectum	51	13	38	
Differentiation				
Well‐moderate	101	26	75	
Poor	49	16	33	.377
TNM stage				
II	83	36	47	
III	67	6	61	<.001
Recurrence				
Without	85	32	53	.003
With	65	10	55	
State of life				
Survival	104	28	76	.659
Death	46	14	32	

Abbreviations: CRC, Colorectal cancer.

### 3.3 Correlation between DEPDC1 expression and CRC patient prognosis

**Figure 3:**
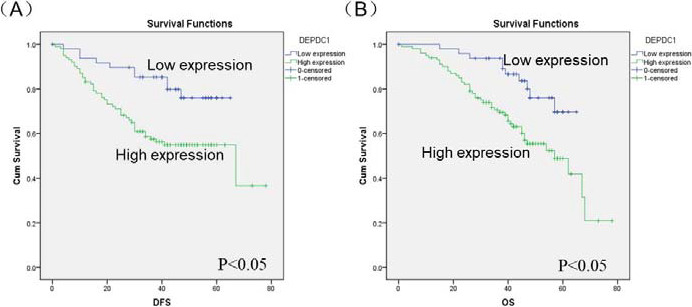
DEPDC1 overexpression predicted poorer DFS and OS in CRC patients. (A) Relationship between DEPDC1 protein expression and DFS. (B) Relationship between DEPDC1 protein expression and OS. CRC, Colorectal cancer; DFS, disease‐free survival; OS, overall survival

By Kaplan‐Meier analysis, disease‐free survival (DFS) and overall survival (OS) curves were calculated based on different DEPDC1 protein levels in CRC. We demonstrated that patients with high DEPDC1 protein expression had a shorter DFS and OS than patients with low DEPDC1 protein expression (*P *< .05, Figure 3). Univariate analysis revealed that the relative level of DEPDC1 protein expression and TNM stage was correlated with DFS (*P *= .005 and *P *= .001, respectively, Table 3), as was the same concerning OS (*P *= .006 and *P *< .001, respectively, Table 3). The other clinicopathological features, such as age, gender, tumor size, tumor location, differentiation, or state of life, were not significant prognosis factors (*P *> .05, Table 3). Multivariate analysis showed that the combination of DEPDC1 protein expression and TNM stage has statistical significance in predicting prognosis (*P* = .024 and *P* = .009, respectively, Table 4).

**Table 3:** Univariate Cox regression model for the survival of CRC patients

Clinicopathological parameters	DFS		OS	
	χ2	*P*	χ2	*P*
Gender	0.425	.514	1.193	.275
Age (y)	0.367	.544	0.949	.330
Location	0.129	.720	0.299	.585
Tumor size	0.277	.599	0.816	.366
Differentiation	0.574	.751	0.409	.815
**TNM stage**	10.196	.001	12.183	<.001
**DEPDC1 expression**	7.939	.005	7.524	.006

Abbreviations: CRC, Colorectal cancer; DFS, disease‐free survival.

**Table 4:** Multivariate Cox regression model for the survival of CRC patients

Clinicopathological parameters	Exp(B)	95% CI for Exp(B)		*P*
		Lower	Upper	
DEPDC1 expression	2.228	1.111	4.468	.024
TNM stage	2.183	1.219	3.909	.009

Note

All statistical tests were two‐sided.

Abbreviations: CI, confidence interval; Exp(B)=OR, odds ratio.

## 4 DISCUSSION

This research aimed to reveal the potential role of DEPDC1 expression in CRC clinical cohorts. In our study, we demonstrated that DEPDC1 expression is strongly correlated with some of the clinicopathological parameters and prognosis in CRC, thereby speculating that DEPDC1 overexpression might contribute to CRC development and the poorer prognosis of CRC patients. So we inferred that DEPDC1 might be considered as a novel biomarker for CRC cancer development and prognosis monitoring.

DEPDC1 overexpression has been found negatively correlated with the prognosis of many malignant tumors.^12^, ^14^, ^15^ Initially, Kanehira et al discovered that DEPDC1 was overexpressed in bladder carcinoma, with no positive expression found in 24 other normal tissues (such as lung, kidney, and ovary) except testis.^12^ Recent study showed that DEPDC1 was overexpressed in breast cancer tissues, with its mRNA level closely related with poor prognosis and progression of breast cancer.^14^ In addition, DEPDC1 has been reported up‐regulated in HCC and correlated with diagnosis as well as poorer prognosis of HCC patients.^15^ In our study, we first detected the expression of DEPDC1 in 150 pairs of CRC and ANCTs by RT‐qPCR and found that the mRNA expression level of DEPDC1 in CRC tissues was significantly higher than that in ANCTs. By IHC, we examined the protein expression of DEPDC1 in 150 CRC tissues and ANCTs and observed that the staining of DEPDC1 was in nucleus of tumor cells, and the proportion of DEPDC1 high expression was significantly greater in CRC compared to that in ANCTs (*P *= .019). These data support the view that DEPDC1 expression levels may be associated with CRC progression. Meanwhile, we assessed the relationship between DEPDC1 protein expression and clinicopathological parameters and concluded that high expression of DEPDC1 in CRC was correlated with TNM stage and recurrence (*P *< .001 and *P *= .003, respectively), while with no significant correlation between DEPDC1 expression and other clinicopathological features (such as age, gender, tumor size, tumor location, differentiation, or state of life, *P *> .05). Furthermore, high DEPDC1 protein expression exhibited poor OS and DFS, and multivariate analysis revealed that the combination of DEPDC1 protein expression and TNM stage has statistical significance in predicting prognosis, indicating that DEPDC1 might be a potential prognostic predictor for CRC in clinical practice. Further studies might verify its prognostic prediction significance in a larger cohort of CRC samples with various histological subtypes.

In addition to correlation with poor prognosis of cancer, numerous studies also explored the biological roles and regulatory mechanisms of DEPDC1 in different malignant tumors. Harada Y et al discovered that DEPDC1 protein inhibits A20 gene expression through binding to ZNF224 (a transcription repressor), leading to IKB‐α protein phosphorylation and degradation, thereby promoting the separation and activation of NF‐κB and IKB‐α, causing its downstream DNA sites of tumor‐related target genes combined to activate oncogenes and induce bladder cancer.^13^ Another research concerning endometrial cancer found that DEPDC1 is involved in cell proliferation and restrain apoptosis of endometrial cancer cell lines, and it promotes tumor growth through the PCDH10‐DEPDC1‐Caspase signal regulation pathway.^19^ Furthermore, DEPDC1 protein has also been found to pass through microtubule‐targeted chemotherapy through the JNK‐dependent pathway, thereby down‐regulating the anti‐apoptotic BCL‐2 family MCL‐1 Protein and inhibiting cell apoptosis.^23^ By inhibiting the expression of DEPDC1, it can inhibit tumor cell growth and promote cell apoptosis.^13^, ^24^ In a recent report concerning CRC, researchers found that the expression level of DEPDC1 in CRC tissues is significantly higher than that in adjacent tissues and plays an oncogenic role in colon cancer cells.^25^ Another study further confirmed that knocking out DEPDC1 significantly inhibits proliferation, migration as well as invasion in colon cancer cells, as well as hinder the epithelial‐mesenchymal transition of intestinal cancer cells. After further exploration, they found that down‐regulating DEPDC1 can reduce zest in intestinal cancer cells. Suppression of zest 12 (SUZ12) protein expression leads to a reduction of trimethylation at lysine 27 of histone H3 (H3K27me3).^26^ It is speculated that DEPDC1 is closely related to the growth and progression of CRC. However, due to few related studies and authoritative reports, its mechanism of action still needs to be further explored and verified. In summary, with the deepening of research on DEPDC1, it has been found that it plays a significant role in the growth and progression of a variety of malignancies and is expected to become one of the new targets for tumor therapy.

## 5 CONCLUSION

Our results showed that DEPDC1 expression is significantly overexpressed in CRC tissues.

Additionally, high expression of DEPDC1 is correlated with poor prognosis of CRC patients and DEPDC1 acts as a prognostic factor for CRC patient survival. Collectively, these findings may provide some evidence for DEPDC1 as a novel prognostic indicator as well as a potential biomarker for assessing CRC.
